# Loneliness and Psychosocial Well-Being in Nursing Homes: A Cross-Sectional Study of Older Adults

**DOI:** 10.3390/healthcare14131873

**Published:** 2026-06-26

**Authors:** Rogelio Hernández-Díaz, Claudia Oteo de Miguel, Alejandra Aguilar-Latorre, Isabel Blasco-González, Mª Rosa Magallón-Botaya

**Affiliations:** 1 Department of Medicine, Psychiatry and Dermatology, Faculty of Medicine, University of Zaragoza, 50009 Zaragoza, Spain; guardiassalud@gmail.com (R.H.-D.); claudiaoteodm@gmail.com (C.O.d.M.); iblasco@salud.aragon.es (I.B.-G.); rosamaga@unizar.es (M.R.M.-B.); 2 Torrero Primary Care Health Center, Aragonese Health Service, 50007 Zaragoza, Spain; 3 Department of Psychology and Sociology, Faculty of Human Sciences and Education, University of Zaragoza, 22003 Huesca, Spain; 4 Institute for Health Research Aragón (IIS Aragón), 50009 Zaragoza, Spain; 5 Research Network on Chronicity, Primary Care and Health Promotion (RICAPPS), Carlos III Health Institute, 28029 Madrid, Spain; 6 Santa Bárbara Primary Care Health Centre, Aragonese Health Service, 22400 Monzón, Spain

**Keywords:** loneliness, older adults, nursing homes, existential loneliness, social loneliness, psychosocial well-being, functional dependence, health literacy

## Abstract

**Highlights:**

**What are the main findings?**

- Social and existential loneliness were moderately related but showed different patterns of association among nursing home residents.
- Activity engagement was consistently associated with lower loneliness, while regular visits were linked mainly to social loneliness and functional independence to existential loneliness.

**What are the implications of the main findings?**

- Nursing homes may consider prioritizing meaningful, socially connective activities as a feasible strategy to address loneliness across different dimensions.
- Care planning may benefit from combining relational contact, functional support, and person-centered engagement to promote psychosocial well-being in long-term care.

**Abstract:**

**Background/Objectives**: Loneliness is a major public health concern in later life and may be especially prevalent among older adults living in nursing homes. Evidence from Spain remains limited regarding modifiable correlates of different loneliness dimensions. This study aimed to describe social and existential loneliness among nursing home residents and examine their associations with sociodemographic, institutional, functional, and psychosocial factors. **Methods**: We conducted a cross-sectional study in Spanish nursing homes using face-to-face structured interviews with residents aged ≥65 years (*n* = 139). Social loneliness was assessed with the ESTE-II scale and existential loneliness with the existential loneliness subscale of the ESTE-R. Functional dependence was measured with the Barthel Index. Health literacy, locus of control, institutional variables, and suicidality-related items were also collected. Spearman correlations and multiple linear regression models with BCa bootstrapped 95% confidence intervals (5000 resamples) were used. **Results**: Social and existential loneliness were moderately correlated (ρ = 0.481, *p* < 0.001). Greater activity engagement was independently associated with lower social (B = −1.105, *p* < 0.001) and existential loneliness (B = −0.732, *p* = 0.029). Receiving visits regularly was associated with lower social loneliness (B = −4.083, *p* = 0.002), but not existential loneliness. Greater functional independence was associated with lower existential loneliness (B = −0.044, *p* = 0.023). **Conclusions**: Activity engagement was a consistent correlate across loneliness dimensions, whereas regular visits were mainly related to social loneliness and functional independence to existential loneliness. These findings support feasible long-term care strategies focused on meaningful activities, relational contact, and functional support.

## 1. Introduction

Loneliness has become a major public health and clinical concern in later life, particularly as populations age and more older adults live with multimorbidity, disability, functional decline, and reduced opportunities for meaningful social participation [1,2]. Loneliness is commonly defined as a distressing subjective experience arising from a perceived discrepancy between desired and actual social relationships, whereas social isolation refers more closely to the objective absence or scarcity of social ties [3,4]. In Spain, the term unwanted loneliness (*soledad no deseada*) is increasingly used to emphasize the involuntary and aversive nature of this experience and its relevance for health, equity, and service planning among older adults [5].

Loneliness and social disconnection have been associated with increased mortality risk, poorer physical health, and adverse mental health outcomes [6,7,8]. In addition, psychiatric literature suggests that emotional processing difficulties, psychological suffering, and suicidal ideation may be interconnected across clinical populations, highlighting the importance of considering loneliness not only as a social condition but also as a potential marker of emotional distress and vulnerability [1,2,9]. Nursing home residents may be especially vulnerable to loneliness. Although long-term care facilities provide safety, continuity of care, and daily support, institutionalization often involves leaving one’s home, neighborhood, routines, and familiar sources of identity [10,11,12].

Residents may also experience bereavement, sensory decline, mobility limitations, functional dependence, reduced privacy, limited autonomy, and disruption of previous social roles [10,11,13]. Importantly, collective living does not necessarily prevent loneliness: residents may be surrounded by others while still lacking emotionally meaningful companionship, reciprocal relationships, or a sense of belonging [11]. Thus, in nursing homes, the quality, reciprocity, and personal significance of relationships may be as important as the frequency of social contact.

Loneliness is increasingly understood as a multidimensional construct. Social loneliness refers mainly to perceived deficits in social integration, companionship, or belonging, whereas existential loneliness is more closely related to feelings of emptiness, meaninglessness, fundamental separateness, and not being deeply understood by others [14]. This distinction may be particularly relevant in nursing homes, where dependence on care staff, functional decline, loss of agency, and disruption of one’s previous life narrative may contribute to existential loneliness even when some social contact is present [10,11,14]. Previous research has documented notable levels of loneliness among residents of long-term care facilities and associations with poorer quality of life and psychosocial well-being [12,15]. Spanish evidence similarly indicates a high burden of social and emotional loneliness among older people living in nursing homes, as well as associations with contextual and health-related factors [16]. However, evidence from Spain remains limited, particularly regarding unwanted loneliness, existential loneliness, and modifiable correlates that can inform feasible routine-care strategies.

Beyond estimating loneliness levels, a clinically relevant question is which potentially modifiable factors are associated with different loneliness dimensions in nursing homes. Institutional and social factors such as length of stay, regular visits, and activity engagement may shape residents’ sense of social connection and belonging. Individual resources, including functional independence, health literacy, and perceived control, may also influence loneliness-related distress, coping, and participation in daily life [17]. Against this background, the present study aimed to (1) describe social loneliness and existential loneliness among institutionalized older adults in Spain; and (2) examine their associations with sociodemographic, institutional, functional, and psychosocial factors relevant to care planning. We expected that greater activity engagement, regular visits, greater functional independence, and better health literacy would be associated with lower loneliness, while also anticipating that social and existential loneliness would show partly distinct patterns of association.

## 2. Materials and Methods

### 2.1. Study Design and Setting

We conducted a descriptive cross-sectional study to examine loneliness in institutionalized older adults living in nursing homes in Spain, operationalized through measures of social loneliness and existential loneliness. Data were collected through face-to-face structured interviews in April 2023.

### 2.2. Participants and Sampling

Participants were recruited using convenience sampling from several nursing homes selected based on feasibility and availability for data collection. The participating facilities included one private nursing home in a semi-rural setting in Soria, one public nursing home in a rural/semi-rural setting in Aragón, one private nursing home in a rural setting in Aragón, and one private nursing home in an urban setting in Zaragoza. Information on staffing characteristics was not systematically collected.

Eligible individuals were current residents aged ≥65 years who were able to understand the interview questions and provide informed consent. Individuals were excluded if they declined participation or presented cognitive or communication impairments that prevented reliable questionnaire completion. No standardized cognitive screening instrument was administered. Instead, eligibility was determined pragmatically through information provided by nursing home staff and the interviewer’s assessment of the resident’s ability to understand the study information, provide informed consent, and complete the face-to-face questionnaire reliably.

An indicative sample size was estimated to contextualize statistical precision under a population-inference scenario for older adults living in long-term care settings in Spain. Assuming a 95% confidence level, 5% precision, and a conservative expected proportion of 0.50, the corresponding sample size would be 384 participants. The achieved sample consisted of 139 nursing home residents, which reflects feasibility constraints inherent to in-facility recruitment and interviewer-administered data collection.

The difference between the estimated sample size for population-level precision and the achieved sample size has implications for statistical power and model stability. In particular, the study may have been underpowered to detect small associations, and multivariable estimates should be interpreted as exploratory and sample-dependent rather than as definitive population-level effects. To reduce overfitting risk, regression models were limited to a small set of a priori covariates, and bootstrapped confidence intervals were used to provide more robust estimates under non-normality.

### 2.3. Procedure and Data Management

Data collection was carried out by a trained interviewer using a single-session, interviewer-administered questionnaire. All participants received verbal and written information about the study aims and procedures and provided written informed consent prior to participation. Responses were coded using non-identifiable numerical codes and stored in a password-protected database. Data were entered into an electronic spreadsheet and subsequently analyzed in SPSS (version 29).

### 2.4. Measures

All variables were collected using a structured data collection form designed for this study.

### 2.5. Primary Variables

Social loneliness was assessed using the ESTE-II Social Loneliness Scale [18], a Spanish instrument developed specifically to assess perceived social loneliness in older adults. The scale was constructed to capture the subjective experience of social loneliness in relation to perceived social support, social participation, and adaptation to contemporary forms of communication. The final version includes 15 items organized into three domains: perceived social support, use of new technologies, and subjective social participation. Items are answered on a three-category scale (always/sometimes/never) and summed to obtain a total score ranging from 0 to 30, with higher scores indicating greater social loneliness. The original documentation reports adequate reliability, with Cronbach’s alpha around 0.72 [18]. For descriptive purposes, social loneliness was categorized as low (0–10), moderate (11–20), and high (21–30). Internal consistency in the present sample was acceptable (Cronbach’s α = 0.757).

Existential loneliness was assessed using the existential loneliness dimension of the ESTE-R scale [19,20]. The ESTE-R is a Spanish-language multidimensional measure of loneliness in older adults that includes family loneliness, marital loneliness, social loneliness, and existential crisis/existential loneliness dimensions. In the present study, we retained the 9-item existential dimension because our objective was to examine the existential component of loneliness separately from social loneliness, and because this dimension was already included in the original data collection protocol. Alternative instruments, such as the De Jong Gierveld Loneliness Scale or the Brief Scale of Existential Loneliness, were not administered and therefore could not be used for the present analyses. Items are rated from 1 (“never”) to 5 (“always”), yielding total scores from 9 to 45, with higher scores indicating greater existential loneliness. In the present sample, internal consistency for this 9-item subscale was low (Cronbach’s α = 0.578). Therefore, findings involving this measure, particularly regression coefficients and effect-size estimates, should be interpreted with caution because measurement error may have attenuated the observed associations.

To facilitate international comparability, the ESTE-II and ESTE-R existential loneliness scores were interpreted in relation to widely used loneliness instruments. Internationally, loneliness is frequently assessed with the UCLA Loneliness Scale, a 20-item unidimensional measure of subjective loneliness and social isolation with strong internal consistency [21], and with the De Jong Gierveld Loneliness Scale, available in 11-item and 6-item versions, which distinguishes emotional and social loneliness [22]. Conceptually, the ESTE-II is closest to the social loneliness component of multidimensional instruments such as the De Jong Gierveld scale, although it includes culturally specific domains such as perceived social support, use of new technologies, and subjective social participation. The ESTE-R existential dimension is less directly comparable with traditional loneliness scales, as existential loneliness has only recently been operationalized in dedicated international measures such as the Brief Scale of Existential Loneliness [23]. Therefore, replication in other countries should consider parallel administration of ESTE-II/ESTE-R with internationally validated instruments to examine convergent validity, measurement equivalence, and cross-cultural comparability.

Functional status in activities of daily living was assessed with the Barthel Index (10 items; total score range 0–100), with higher scores indicating greater functional independence [24]. In the present sample, internal consistency was high (Cronbach’s α = 0.899).

### 2.6. Secondary Variables

Sociodemographic variables included age (years), sex (female/male), and educational level (no formal education/primary/secondary/university).

Institutional and social variables included length of institutionalization (recorded in days), receiving visits regularly (yes/no), visit frequency (days between visits), and number of activities the participant reported engaging in within the nursing home (count).

Health literacy was assessed using six selected items from the HLS-EU-Q16 [25]. These items were selected during questionnaire design to provide a brief pragmatic indicator of residents’ perceived difficulty in accessing, understanding, appraising, and using health-related information, while reducing respondent burden in face-to-face interviews with older nursing home residents. Each item was rated on a 4-point difficulty scale from 1 (“very easy”) to 4 (“very difficult”), and item scores were summed, with higher scores indicating greater perceived difficulty and therefore lower health literacy [26]. Because only six items were administered, this score should not be interpreted as the full HLS-EU-Q16 index or used to derive the standard HLS-EU-Q16 categories. Internal consistency in the present sample was acceptable (Cronbach’s α = 0.783), but comparability with studies using the complete HLS-EU-Q16 is limited.

Locus of control was measured with a single item assessing agreement with the statement “I feel that what happens in my life is often determined by factors outside my control,” rated from 1 (strongly disagree) to 6 (strongly agree), with higher scores indicating a more external locus of control [27]. This item was included as a brief pragmatic indicator of perceived external control in order to reduce respondent burden during face-to-face interviews with older nursing home residents. However, because locus of control is a multidimensional construct and was assessed with a single item rather than a validated multi-item scale, findings involving this variable should be interpreted as exploratory.

Suicidality-related items were assessed using three study-specific lifetime dichotomous questions designed to identify whether participants had ever experienced: (1) feeling that life was not worth living, (2) thoughts about taking their own life without actual intent, and (3) a suicide attempt. These items were included as brief exploratory indicators of lifetime self-harm-related experiences and psychological distress, rather than as a standardized suicidality assessment. They were formulated in a simple yes/no format to reduce respondent burden and facilitate administration during face-to-face interviews with older nursing home residents. No formal psychometric validation was available for these items, and they should not be interpreted as equivalent to validated instruments assessing current suicidal ideation or suicide risk.

For binary yes/no variables, regular visits, previous stay in another nursing home, and suicidality-related items were coded as 1 = yes and 0 = no. Sex and educational level were coded numerically for analysis. Length of stay was recorded in days, and visit frequency was expressed as the number of days between visits. Activities were analyzed as the total number of activities reported by each resident. Higher health literacy scores indicated lower health literacy, and higher locus of control scores indicated a more external locus of control.

### 2.7. Statistical Analysis

Analyses were performed using SPSS version 29 [28]. Internal consistency was assessed with Cronbach’s alpha for multi-item measures (Barthel Index, health literacy items, ESTE-II, and the ESTE-R existential loneliness 9-item subscale).

Normality of continuous variables was evaluated using the Kolmogorov–Smirnov test. Given non-normal distributions, non-parametric procedures were used for bivariate analyses. Categorical variables are reported as frequencies and percentages. Continuous variables are summarized using the mean and standard deviation, as well as the median. Associations between study variables were examined using Spearman’s rank correlation coefficient (ρ) with 95% confidence intervals (Fisher’s *z* method).

To examine independent correlates of loneliness beyond bivariate associations, we fitted multiple linear regression models for social loneliness (ESTE-II total) and existential loneliness (ESTE-R existential loneliness), including a priori covariates (age, sex, education, length of institutionalization, regular visits, activity engagement, health literacy, and functional dependence). Given non-normality, regression coefficients were estimated with bias-corrected and accelerated (BCa) bootstrapped 95% confidence intervals based on 5000 resamples. Regression analyses were conducted using complete cases (*n* = 134). Collinearity was assessed using tolerance and variance inflation factors (VIF). Statistical significance was set at *p* < 0.05 (two-tailed).

### 2.8. Ethics

The study protocol was approved by the Regional Research Ethics Committee (CEICA; C.I. PI23/146, approval 5 April 2023). All participants provided written informed consent. No directly identifying personal data were collected, and data were processed in accordance with applicable data protection regulations.

## 3. Results

### 3.1. Sample Characteristics and Descriptive Findings

A total of 139 institutionalized older adults participated. Most participants were women (59.7%), and the most common age group was 81–90 years (44.6%). The mean age was 84.81 years (*SD* = 7.75; median = 86). Education was predominantly primary level (67.6%).

Regarding social contact, most participants reported receiving visits regularly (84.9%). The average interval between visits was 13.77 days (*SD* = 23.57; median = 7). Participants reported a mean of 2.01 activities (*SD* = 1.28; median = 2).

Functional status indicated mainly mild dependence: the mean Barthel Index score was 72.00 (*SD* = 25.10; median = 77), with almost half categorized as mild dependence (60–99). Loneliness scores were in the mid-range: ESTE-R existential loneliness mean = 21.95 (*SD* = 5.25; median = 22) and ESTE-II social loneliness mean = 11.70 (*SD* = 4.69; median = 12). Categorical distributions of loneliness levels are shown in Table 1.

In relation to self–harm indicators, 38.8% reported having felt at some point that life was not worth living, 11.5% reported having had suicidal thoughts (without intent), and 4.3% reported a lifetime suicide attempt (Table 1 and Table 2).

### 3.2. Correlational Analysis

Spearman correlations (Table 3) showed that social loneliness (ESTE–II) was strongly and positively associated with existential loneliness (ESTE–R) (ρ = 0.481, *p* < 0.001). Higher loneliness scores were also associated with lower activity engagement (ESTE–R with activities: ρ = −0.221, *p* = 0.009; ESTE–II with activities: ρ = −0.308, *p* < 0.001) and worse health literacy (ESTE–R: ρ = 0.196, *p* = 0.021; ESTE–II: ρ = 0.252, *p* = 0.003). Greater existential loneliness (ESTE–R) was additionally associated with greater functional dependence (Barthel with ESTE–R: ρ = −0.221, *p* = 0.009). Social loneliness was also associated with a more external locus of control (ρ = 0.212, *p* = 0.029).

Institutional factors were relevant: length of stay was associated with longer intervals between visits (ρ = 0.239, *p* = 0.009) and with higher social loneliness (ρ = 0.265, *p* = 0.002). In addition, receiving visits was associated with lower social loneliness (ρ = −0.320, *p* < 0.001). Regarding suicidality-related items, higher social and existential loneliness scores were associated with a greater likelihood of reporting that life had not been worth living and having had lifetime suicidal thoughts. Lifetime suicide attempt was not significantly associated with either loneliness measure.

The full correlation matrix, including non-significant correlations, is available in Supplementary Table S1.

To examine independent correlates of social loneliness (ESTE-II total) beyond bivariate associations, we fitted a multiple linear regression model with BCa bootstrapped 95% confidence intervals (5000 resamples). The model was statistically significant, *F*(8, 125) = 6.55, *p* < 0.001, explaining 29.5% of the variance (*R*^2^ = 0.295; adjusted *R*^2^ = 0.250).

In the bootstrapped model, receiving visits regularly (1 = yes, 0 = no) and number of activities were independently associated with lower social loneliness. Specifically, receiving visits was independently associated with lower ESTE-II scores (*B* = −4.08, BCa 95% CI [−6.64, −1.46], *p* = 0.002), and participating in more activities was associated with lower loneliness (*B* = −1.11, BCa 95% CI [−1.63, −0.55], *p* < 0.001). Worse health literacy was also associated with higher loneliness (*B* = 0.27, BCa 95% CI [0.006, 0.524], *p* = 0.043). Age, sex, education, length of stay, and functional independence (Barthel Index) were not significant predictors in the bootstrapped model. Collinearity was low (VIFs ≈ 1.05–1.20) (Table 4).

A second multiple linear regression model (with BCa bootstrapped 95% confidence intervals; 5000 resamples) examined independent correlates of existential loneliness (ESTE-R existential loneliness). The model was significant, *F*(8, 125) = 2.76, *p* = 0.008, explaining 15.0% of the variance (*R*^2^ = 0.150; adjusted *R*^2^ = 0.096).

In the bootstrapped model, activity engagement and functional independence were significant independent predictors. Specifically, a higher number of activities was associated with lower ESTE-R scores (*B* = −0.73, BCa 95% CI [−1.39, −0.10], *p* = 0.029), and higher Barthel Index scores (greater independence) were associated with lower existential loneliness (*B* = −0.044, BCa 95% CI [−0.081, −0.008], *p* = 0.023). Regular visits (1 = yes, 0 = no) were not independently associated with ESTE-R scores in this model (*p* = 0.186). Collinearity was low (VIFs ≈ 1.05–1.20) (Table 5).

## 4. Discussion

This study provides updated evidence on loneliness-related experiences among institutionalized older adults in Spain, highlighting both the multidimensionality of loneliness and several potentially modifiable correlates relevant to long-term care practice. Overall, levels of social loneliness and existential loneliness were in the mid-range, and both dimensions were moderately interrelated, supporting the idea that loneliness in later life is not a single construct but includes separable social, emotional, and existential components [4,6,14]. This distinction is particularly important in nursing home environments, where residents may live in close physical proximity to others while still lacking meaningful companionship, reciprocal relationships, autonomy, continuity of identity, or a sense of being deeply understood [10,11,15]. Therefore, the co-occurrence observed in this study should not be interpreted as redundancy between the two measures, but rather as evidence that social and existential loneliness may overlap while reflecting different forms of suffering in institutional care.

Although several bivariate associations reached statistical significance, many of the Spearman correlations were small to moderate in magnitude and should therefore be interpreted cautiously. Similarly, the regression models explained a moderate proportion of variance in social loneliness and a more limited proportion of variance in existential loneliness, particularly in the existential loneliness model (adjusted R^2^ = 0.096). These findings suggest that the variables included in the study captured only part of the complexity of loneliness among nursing home residents, and that other unmeasured factors—such as quality of relationships, depressive symptoms, cognitive status, personality traits, facility-level characteristics, previous life experiences, or residents’ perceived autonomy and meaning in daily life—may also play an important role. Accordingly, both the bivariate and multivariable findings should be interpreted as exploratory associations rather than as comprehensive explanatory models.

In the multivariable model for social loneliness (ESTE-II), receiving visits regularly and participating in more activities were independently associated with lower loneliness. This pattern aligns with a large body of research suggesting that social contact and engagement opportunities are central protective factors against loneliness in residential care [12,15]. Importantly, the association with visits remained after adjusting for age, sex, education, length of stay, health literacy, and functional dependence, suggesting that routine relational contact may have an independent role beyond residents’ health status or time spent in the institution.

In the context of nursing homes, this finding is clinically relevant because social loneliness may persist even in communal settings. Residents may share spaces, meals, and scheduled activities with others, but these interactions do not necessarily provide emotionally meaningful companionship or a sense of belonging. Regular visits may therefore operate not only as a source of social contact, but also as a bridge with residents’ previous lives, family roles, and personal histories. This may help explain why visits were independently associated with social loneliness in the present study, whereas their association with existential loneliness was weaker after adjustment. The result reinforces the importance of preserving relational continuity between residents and their external social networks, rather than assuming that institutional cohabitation is sufficient to meet social needs [11,15,16].

These findings also resonate with evidence from the COVID-19 period, when visitation restrictions highlighted the psychosocial importance of family contact for nursing home residents [29,30]. Although the present study was not designed to assess pandemic-related effects, the results support care practices that facilitate meaningful contact through in-person visits when possible and supported alternatives, such as phone or video calls, when barriers exist [31].

The independent association between activity engagement and lower social loneliness is also consistent with intervention research and broader evidence syntheses suggesting that group-based and socially meaningful activities can reduce loneliness and improve social connectedness in older adults [32,33]. In nursing homes specifically, recent work emphasizes that interventions often vary in effectiveness depending on whether they target mechanisms such as relationship formation, shared identity, and sustained social roles—highlighting the need to design activities that are not merely “occupational” but socially connective and personally meaningful [34].

In contrast, the multivariable model for existential loneliness indicated that functional independence (Barthel Index) and activity engagement were the key independent correlates, whereas regular visits did not remain significant after adjustment. This divergence across outcomes is conceptually coherent, but it should be interpreted cautiously because the ESTE-R existential loneliness subscale showed low internal consistency in the present sample. Measurement error may have attenuated associations and reduced the stability of estimates involving existential loneliness. Therefore, findings for existential loneliness should be regarded as exploratory and in need of replication with psychometrically stronger measures. Existential loneliness in nursing homes may be less dependent on the mere availability of social contact and more closely related to residents’ experiences of dependence, loss of agency, disruption of previous routines, and changes in identity and purpose [10,11,14]. Functional decline can limit residents’ capacity to move freely, choose how to spend their time, initiate interactions, and participate in valued activities. In this sense, dependence may not only reduce practical autonomy but also intensify the feeling that one’s life is being organized by others, which may contribute to existential loneliness.

The fact that regular visits were not independently associated with existential loneliness after adjustment should not be interpreted as meaning that family contact is unimportant. Rather, it suggests that existential loneliness may require forms of support that go beyond contact frequency. Residents may receive visits and still feel existentially lonely if they perceive a loss of meaning, usefulness, dignity, or continuity with their previous self. This interpretation is consistent with qualitative and conceptual work indicating that existential well-being in nursing homes is closely connected to autonomy, recognition, meaningful relationships, and opportunities to remain a person with a valued history and role, rather than only a recipient of care [10,11,14].

The protective association of activities across both loneliness dimensions suggests a potentially important cross-cutting mechanism: activities can facilitate social connection, structure time, support identity, and reinforce a sense of purpose. This is consistent with intervention evidence indicating that approaches combining engagement and social participation—rather than purely informational or passive programming—are more likely to influence loneliness outcomes [32,33]. Taken together, these findings imply that care planning should consider both relationship-based strategies (supporting visits and relational continuity) and capability-based strategies (maintaining independence and enabling meaningful engagement). In nursing home settings, such activities may be especially relevant when they allow residents to make choices, maintain previous interests, contribute to group life, and be recognized by others beyond their status as care recipients.

Given the scoring of the health literacy variable, higher scores indicated lower health literacy and were modestly associated with higher social loneliness. This finding should be interpreted cautiously, as only six selected HLS-EU-Q16 items were used and the observed association was small. Nevertheless, health literacy may be relevant in institutional care because it can influence how residents understand services, communicate needs, and participate in health-related decisions. Rather than supporting specific health literacy interventions on its own, this exploratory finding suggests that communication accessibility may be a useful area for future research and quality-improvement initiatives.

A nontrivial proportion of participants reported having felt that life was not worth living, as well as lifetime suicidal thoughts or attempts. The bivariate associations between loneliness measures and suicidality-related items are consistent with previous syntheses suggesting links between loneliness and suicidal ideation in older adults [1,15]. However, these findings should be interpreted cautiously. The suicidality-related variables were assessed using study-specific lifetime dichotomous items rather than standardized instruments, and they did not capture current suicidal ideation, severity, intent, planning, or clinical risk. In addition, the cross-sectional design does not permit causal inference, and the low frequency of suicide attempts limited more complex modeling. Therefore, the observed associations should be considered exploratory and should not be interpreted as evidence that loneliness caused suicidal thoughts or behaviors. Even so, these findings underscore the clinical relevance of assessing loneliness alongside broader indicators of emotional distress, especially in institutional settings where residents may experience losses, transitions, and restricted autonomy [2]. Future prospective studies using validated suicidality measures are needed to clarify temporal relationships and clinical risk pathways in institutionalized older adults.

From a practice perspective, these findings point to several feasible and actionable directions for long-term care. First, facilities should strengthen and diversify contact pathways. While enabling regular in-person visits remains a key priority, homes can also proactively identify residents with limited or irregular contact and offer supported alternatives, such as assisted phone or video calls and structured family involvement plans. Evidence from the pandemic period has shown both the psychosocial harms of restricted contact and the potential value of alternative visitation modalities when in-person visits are constrained [29,31].

Second, nursing homes should invest in meaningful, socially connective activities. Because activity engagement was associated with lower loneliness across dimensions, programs are likely to be most beneficial when they prioritize continuity, resident choice, and social mechanisms that foster relationships, belonging, and identity—rather than focusing only on passive or purely recreational programming [32,34]. In practice, this can translate into activity design that supports repeated group membership, peer connection, and opportunities for residents to contribute or take on valued roles. This distinction is important because activities that merely occupy time may have limited impact, whereas activities that promote choice, reciprocity, contribution, and continuity of identity may address both social and existential dimensions of loneliness.

Third, the results support efforts to integrate function-oriented approaches with psychosocial care. Given that functional dependence was independently associated with existential loneliness, interventions that maintain mobility and independence where possible—through rehabilitation, tailored physical activity, and environmental adaptations—may help address deeper loneliness-related suffering. Multicomponent approaches that combine physical capability with opportunities for social participation may be especially promising in residential care settings [13].

Finally, communication accessibility may be considered as a supportive component of person-centered care. Although the association between health literacy and loneliness was modest and exploratory, strategies such as plain language, repetition, visual aids, and structured opportunities for questions may help residents engage with care processes and participation opportunities. Future studies should examine whether such approaches can contribute to psychosocial well-being in nursing home settings.

### 4.1. Strengths and Limitations

This study’s strengths include an updated dataset with a consolidated sample, assessment of two theoretically meaningful loneliness-related dimensions (social loneliness and existential loneliness), and the use of bootstrapped confidence intervals to improve robustness under non-normal distributions. The combination of bivariate and multivariable analyses adds interpretability by distinguishing crude from adjusted associations.

Several limitations should be considered. First, the cross-sectional design precludes causal conclusions; for example, lower activity engagement may contribute to loneliness, but loneliness may also reduce motivation to participate. Second, the use of convenience sampling from four nursing homes, together with the absence of systematically collected facility-level information such as staffing characteristics, limits external validity and restricts the generalizability of the findings to other long-term care settings. Third, the achieved sample size was smaller than the estimated size required for population-level precision, which may have limited statistical power to detect small associations and may affect the stability of multivariable regression estimates. Accordingly, the regression findings should be interpreted as exploratory and sample-dependent. Fourth, cognitive and communication eligibility was determined pragmatically rather than through a standardized cognitive screening instrument, which may have introduced variability in participant selection and limits comparability with studies using formal cognitive inclusion criteria. Fifth, measures relied on self-report and interviewer administration, which may have introduced interviewer effects, response bias, and social desirability bias, particularly for sensitive topics such as loneliness and suicidality-related experiences. Sixth, the existential loneliness item set showed low internal consistency, suggesting measurement error that could attenuate associations and indicating a need for careful interpretation of that construct. Seventh, health literacy was assessed using six selected items from the HLS-EU-Q16 rather than the complete instrument; therefore, the score should be interpreted as a brief study-specific indicator, and comparability with studies using the full HLS-EU-Q16 is limited. Eighth, the single-item assessment of locus of control may have reduced reliability and construct validity, and the modest association with social loneliness should therefore be interpreted cautiously. Future studies should use validated multi-item locus of control instruments. Ninth, suicidality-related experiences were assessed using three study-specific lifetime dichotomous items rather than validated suicidality instruments. These items provided exploratory information on lifetime self-harm-related experiences but did not assess current suicidal ideation, severity, intent, planning, or clinical risk. Therefore, the findings involving these variables should be interpreted cautiously and require replication in prospective studies using standardized suicidality measures. Finally, because the ESTE-II and ESTE-R scales are mainly documented in Spanish-language literature, future studies should further examine their cross-cultural validity and comparability with internationally used loneliness measures, particularly the UCLA Loneliness Scale, the De Jong Gierveld Loneliness Scale, and emerging measures of existential loneliness. Some variables also had missing data, and regression models were based on complete cases, which may have introduced selection effects if missingness was not random.

### 4.2. Future Research

Future work should prioritize longitudinal designs to clarify directionality and identify trajectories of loneliness after institutionalization, including how loneliness evolves with changes in function, social contact, and engagement opportunities. Intervention studies in nursing homes should explicitly specify the mechanisms through which programs aim to reduce loneliness (e.g., enhancing reciprocity, belonging, shared identity), as recommended by recent reviews [34]. Given the apparent importance of activity engagement, pragmatic trials comparing activity formats (resident-led vs. staff-led; group vs. dyadic; purpose-oriented vs. recreational) would be valuable. Research should also examine how communication and health literacy supports interact with psychosocial interventions, and whether improving accessibility and participation pathways can reduce loneliness and related distress. Finally, because visitation patterns and policies can shift with public health circumstances, evaluating flexible and hybrid contact models (in-person plus technology-supported engagement) remains an important applied priority [29,31].

## 5. Conclusions

This cross-sectional study suggests that loneliness among nursing home residents is multidimensional and associated with potentially modifiable factors. Activity engagement was consistently related to lower social and existential loneliness, while regular visits were mainly associated with lower social loneliness and functional independence with lower existential loneliness. These findings support care strategies that combine meaningful activities, relational contact, and functional support in long-term care settings. Given the observational design and measurement limitations, longitudinal and intervention studies are needed to clarify causal pathways.

## Figures and Tables

**Table 1:** Sample characteristics and categorical study variables (*n* = 139).

Characteristic	Category	*n*	%
Sex	Male	56	40.3
	Female	83	59.7
Age group (years)	65–80	41	29.5
	81–90	62	44.6
	91–100	36	25.9
Education level	No formal education	16	11.5
	Primary	94	67.6
	Secondary	22	15.8
	University	7	5.0
Previous stay in another nursing home	Yes	35	25.2
	No	104	74.8
Receives visits regularly	Yes	118	84.9
	No	20	14.4
Barthel Index categories	Total dependence (<20)	6	4.3
	Severe dependence (20–35)	4	2.9
	Moderate dependence (40–55)	28	20.1
	Mild dependence (60–99)	69	49.6
	Independent (100)	32	23.0
ESTE–R existential loneliness level	Low	63	45.3
	Moderate	73	52.5
	High	3	2.2
ESTE–II social loneliness level	Low	63	45.3
	Moderate	67	48.2
	High	8	5.8
Locus of control (item response)	Strongly disagree	3	2.2
	Disagree	22	15.8
	Somewhat disagree	18	12.9
	Somewhat agree	19	13.7
	Agree	29	20.9
	Strongly agree	17	12.2
Felt life was not worth living (lifetime)	Yes	54	38.8
	No	85	61.2
Thought about suicide (lifetime)	Yes	16	11.5
	No	123	88.5
Attempted suicide (lifetime)	Yes	6	4.3
	No	133	95.7

Note. Some categorical variables show minor missingness: receives visits regularly and ESTE–II level sum to *n* = 138; locus of control item responses sum to *n* = 108.

**Table 2:** Descriptive statistics for continuous study variables.

Variable	M	Median	SD
Age (years)	84.81	86	7.75
Length of stay (days)	1001.66	498	1565.25
Days between visits	13.77	7	23.57
Barthel Index (0–100)	72.00	77	25.10
ESTE–R existential loneliness (9–45)	21.95	22	5.25
ESTE–II social loneliness (0–30)	11.70	12	4.69
Activities (count)	2.01	2	1.28
Health literacy score (higher = worse)	11.53	12	2.75
Locus of control (1–6; higher = external)	3.93	4	1.46

Note. M = mean; SD = standard deviation; ESTE-II = Social Loneliness Scale; ESTE-R = Revised ESTE Loneliness Scale.

**Table 3:** Significant Spearman correlations with 95% confidence intervals (*n* = 139).

Variable 1	Variable 2	ρ	*p*	95% CI
Age	Education	−0.232	0.006	[−0.388, −0.064]
Age	Regular visits	0.277	0.001	[0.110, 0.428]
Age	Barthel Index	−0.170	0.046	[−0.331, 0.002]
Age	Lifetime suicide attempt	−0.203	0.016	[−0.362, −0.033]
Sex	Length of stay	−0.180	0.036	[−0.342, −0.007]
Sex	Regular visits	0.169	0.047	[−0.003, 0.332]
Sex	Activities	0.205	0.016	[0.034, 0.364]
Length of stay	Regular visits	−0.266	0.002	[−0.420, −0.097]
Length of stay	Days between visits	0.239	0.009	[0.056, 0.406]
Length of stay	Social loneliness (ESTE–II)	0.265	0.002	[0.097, 0.419]
Prior nursing home stay	Social loneliness (ESTE–II)	−0.197	0.020	[−0.357, −0.026]
Regular visits	Social loneliness (ESTE–II)	−0.320	<0.001	[−0.467, −0.156]
Regular visits	Lifetime suicidal thoughts	−0.172	0.043	[−0.334, 0.000]
Barthel Index	Existential loneliness (ESTE–R)	−0.221	0.009	[−0.378, −0.051]
Existential loneliness (ESTE–R)	Social loneliness (ESTE–II)	0.481	<0.001	[0.337, 0.603]
Existential loneliness (ESTE–R)	Activities	−0.221	0.009	[−0.379, −0.051]
Existential loneliness (ESTE–R)	Health literacy	0.196	0.021	[0.026, 0.356]
Existential loneliness (ESTE–R)	Life not worth living	0.239	0.005	[0.071, 0.394]
Existential loneliness (ESTE–R)	Lifetime suicidal thoughts	0.192	0.024	[0.021, 0.351]
Social loneliness (ESTE–II)	Activities	−0.308	<0.001	[−0.456, −0.143]
Social loneliness (ESTE–II)	Health literacy	0.252	0.003	[0.084, 0.407]
Social loneliness (ESTE–II)	Locus of control	0.212	0.029	[0.017, 0.391]
Social loneliness (ESTE–II)	Life not worth living	0.236	0.005	[0.067, 0.392]
Social loneliness (ESTE–II)	Lifetime suicidal thoughts	0.219	0.010	[0.049, 0.377]
Activities	Locus of control	−0.196	0.043	[−0.377, −0.001]
Locus of control	Lifetime suicidal thoughts	0.207	0.032	[0.013, 0.386]

Note. ρ = Spearman’s rank correlation coefficient. CI = confidence interval; ESTE-II = Social Loneliness Scale; ESTE-R = Revised ESTE Loneliness Scale. Confidence intervals are based on Fisher’s z. Only statistically significant correlations are shown. Binary yes/no variables were coded as 1 = yes and 0 = no.

**Table 4:** Multiple linear regression predicting social loneliness (ESTE-II total) with BCa bootstrapped 95% confidence intervals (*n* = 134).

Predictor	B	SE	β	*p* (Bootstrap)	95% CI (BCa)
Age (years)	0.022	0.049	0.036	0.645	[−0.082, 0.122]
Sex	−0.577	0.765	−0.060	0.454	[−2.064, 0.908]
Education	−0.670	0.544	−0.095	0.191	[−1.680, 0.346]
Length of stay (days)	0.00008	0.000	0.027	0.778	[0.000, 0.001]
**Regular visits (1 = yes, 0 = no)**	−4.083	1.084	−0.310	**0** **.002**	[−6.637, −1.461]
**Activities (count)**	−1.105	0.287	−0.301	**<0.001**	[−1.634, −0.549]
**Health literacy (higher = worse)**	0.274	0.139	0.155	**0** **.043**	[0.006, 0.524]
Barthel Index (0–100)	−0.027	0.015	−0.144	0.077	[−0.059, 0.006]

Note. Bold formatting indicates statistically significant predictors (*p* < 0.05). Dependent variable: ESTE-II total score, with higher scores indicating greater social loneliness. B = unstandardized coefficient; SE = standard error; β = standardized coefficient; BCa = bias-corrected and accelerated; CI = confidence interval. Confidence intervals are based on 5000 bootstrap samples. Model fit: R = 0.543, R^2^ = 0.295, adjusted R^2^ = 0.250; F(8, 125) = 6.55, *p* < 0.001. Collinearity was low (VIF range ≈ 1.05–1.20).

**Table 5:** Multiple linear regression predicting Existential loneliness (ESTE-R existential loneliness) with BCa bootstrapped 95% confidence intervals (*n* = 134).

Predictor	B	SE	β	*p* (Bootstrap)	95% CI (BCa)
Age (years)	−0.008	0.060	−0.012	0.898	[−0.130, 0.112]
Sex	−0.193	0.934	−0.018	0.844	[−2.065, 1.637]
Education	−0.802	0.664	−0.102	0.148	[−1.946, 0.452]
Length of stay (days)	0.000	0.000	−0.122	0.179	[−0.001, 0.001]
Regular visits (1 = yes, 0 = no)	−2.079	1.324	−0.142	0.186	[−5.126, 0.799]
**Activities (count)**	−0.732	0.351	−0.179	**0** **.029**	[−1.386, −0.101]
Health literacy (higher = worse)	0.249	0.170	0.127	0.097	[−0.074, 0.561]
**Barthel Index (0–100)**	−0.044	0.018	−0.208	**0** **.023**	[−0.081, −0.008]

Note. Bold formatting indicates statistically significant predictors (*p* < 0.05). Dependent variable: ESTE-R existential loneliness score, with higher scores indicating greater existential loneliness. B = unstandardized coefficient; SE = standard error; β = standardized coefficient; BCa = bias-corrected and accelerated; CI = confidence interval. Confidence intervals are based on 5000 bootstrap samples. Model fit: R = 0.387, R^2^ = 0.150, adjusted R^2^ = 0.096; F(8, 125) = 2.76, *p* = 0.008. Collinearity was low (VIF range ≈ 1.05–1.20).

## Data Availability

The data presented in this study are available on request from the corresponding author [A.A.-L.] due to privacy and ethical restrictions. The data are not publicly available because they contain sensitive information from older adults living in nursing homes.

## Supplementary Materials

The following supporting information can be downloaded at: https://www.mdpi.com/article/10.3390/healthcare14131873/s1, Table S1: Full Spearman correlations with 95% confidence intervals for study variables.

## Abbreviations

The following abbreviations are used in this manuscript:

BCa	Bias-corrected and accelerated
CI	Confidence interval
ESTE-II	Social Loneliness Scale
ESTE-R	Revised ESTE Loneliness Scale
HLS-EU-Q16	European Health Literacy Survey Questionnaire, 16-item version
VIF	Variance inflation factor
