# Supplementary material for: Loneliness and Psychosocial Well-Being in Nursing Homes: A Cross-Sectional Study of Older Adults
Source: Healthcare (Basel). 2026 Jun 26;14(13):1873. doi: 10.3390/healthcare14131873 (PMC13362440; doi:10.3390/healthcare14131873)
Supplement: Supplementary file 1 [file healthcare-14-01873-s001.zip › healthcare-4330053-supplementary.pdf]

Supplementary Table S1. Full Spearman correlations with 95% confidence intervals for study variables

Variable 1	Variable 2	ρ	p	95% CI
Age	Sex	.059	.489	[−0.113, 0.228]
Age	Education	−.232	.006	[−0.388, −0.064]
Age	Length of stay	.168	.050	[−0.005, 0.332]
Age	Prior nursing home stay	.081	.345	[−0.092, 0.249]
Age	Regular visits	.277	.001	[0.110, 0.428]
Age	Days between visits	−.131	.152	[−0.306, 0.054]
Age	Barthel Index	−.170	.046	[−0.331, 0.002]
Age	Existential loneliness (ESTE−R)	−.053	.535	[−0.222, 0.119]
Age	Social loneliness (ESTE−II)	−.013	.876	[−0.185, 0.159]
Age	Activities	.034	.690	[−0.139, 0.205]
Age	Health literacy	−.083	.333	[−0.250, 0.090]
Age	Locus of control	−.071	.463	[−0.262, 0.125]
Age	Life not worth living	.045	.601	[−0.128, 0.214]
Age	Lifetime suicidal thoughts	−.136	.112	[−0.300, 0.037]
Age	Lifetime suicide attempt	−.203	.016	[−0.362, −0.033]
Sex	Education	.054	.527	[−0.118, 0.223]
Sex	Length of stay	−.180	.036	[−0.342, −0.007]
Sex	Prior nursing home stay	.064	.453	[−0.108, 0.233]
Sex	Regular visits	.169	.047	[−0.003, 0.332]
Sex	Days between visits	−.074	.420	[−0.253, 0.111]
Sex	Barthel Index	−.149	.080	[−0.313, 0.023]
Sex	Existential loneliness (ESTE−R)	−.043	.611	[−0.213, 0.129]
Sex	Social loneliness (ESTE−II)	−.160	.061	[−0.323, 0.012]
Sex	Activities	.205	.016	[0.034, 0.364]
Sex	Health literacy	.058	.496	[−0.114, 0.227]
Sex	Locus of control	−.027	.782	[−0.220, 0.168]
Sex	Life not worth living	.053	.537	[−0.120, 0.222]
Sex	Lifetime suicidal thoughts	.066	.437	[−0.106, 0.235]
Sex	Lifetime suicide attempt	.102	.231	[−0.070, 0.269]
Education	Length of stay	−.125	.149	[−0.291, 0.050]
Education	Prior nursing home stay	.063	.464	[−0.110, 0.231]
Education	Regular visits	−.033	.704	[−0.203, 0.140]
Education	Days between visits	.036	.691	[−0.148, 0.218]
Education	Barthel Index	.148	.082	[−0.024, 0.311]
Education	Existential loneliness (ESTE−R)	−.122	.153	[−0.287, 0.050]
Education	Social loneliness (ESTE−II)	−.144	.093	[−0.308, 0.029]
Education	Activities	.060	.484	[−0.113, 0.230]
Education	Health literacy	−.096	.263	[−0.263, 0.077]
Education	Locus of control	−.016	.868	[−0.210, 0.179]
Education	Life not worth living	−.027	.753	[−0.197, 0.145]
Education	Lifetime suicidal thoughts	−.019	.821	[−0.190, 0.152]
Education	Lifetime suicide attempt	.020	.813	[−0.152, 0.191]
Length of stay	Prior nursing home stay	.032	.715	[−0.142, 0.204]
Length of stay	Regular visits	−.266	.002	[−0.420, −0.097]
Length of stay	Days between visits	.239	.009	[0.056, 0.406]
Length of stay	Barthel Index	.006	.944	[−0.167, 0.179]
Length of stay	Existential loneliness (ESTE−R)	.066	.444	[−0.108, 0.237]
Length of stay	Social loneliness (ESTE−II)	.265	.002	[0.097, 0.419]
Length of stay	Activities	−.043	.623	[−0.215, 0.132]
Length of stay	Health literacy	−.074	.392	[−0.244, 0.100]
Length of stay	Locus of control	.042	.672	[−0.156, 0.236]
Length of stay	Life not worth living	.084	.332	[−0.091, 0.253]

Variable 1	Variable 2	ρ	p	95% CI
Length of stay	Lifetime suicidal thoughts	.010	.909	[−0.164, 0.183]
Length of stay	Lifetime suicide attempt	−.024	.780	[−0.197, 0.150]
Prior nursing home stay	Regular visits	−.003	.968	[−0.175, 0.169]
Prior nursing home stay	Days between visits	−.125	.170	[−0.301, 0.059]
Prior nursing home stay	Barthel Index	.086	.313	[−0.086, 0.254]
Prior nursing home stay	Existential loneliness (ESTE−R)	−.089	.299	[−0.256, 0.084]
Prior nursing home stay	Social loneliness (ESTE−II)	−.197	.020	[−0.357, −0.026]
Prior nursing home stay	Activities	.102	.234	[−0.071, 0.269]
Prior nursing home stay	Health literacy	−.061	.473	[−0.230, 0.111]
Prior nursing home stay	Locus of control	−.046	.634	[−0.239, 0.149]
Prior nursing home stay	Life not worth living	.020	.812	[−0.152, 0.191]
Prior nursing home stay	Lifetime suicidal thoughts	.001	.986	[−0.170, 0.173]
Prior nursing home stay	Lifetime suicide attempt	−.040	.641	[−0.210, 0.132]
Regular visits	Days between visits	−.089	.329	[−0.269, 0.096]
Regular visits	Barthel Index	−.068	.431	[−0.237, 0.106]
Regular visits	Existential loneliness (ESTE−R)	−.135	.114	[−0.300, 0.038]
Regular visits	Social loneliness (ESTE−II)	−.320	<.001	[−0.467, −0.156]
Regular visits	Activities	.101	.241	[−0.073, 0.269]
Regular visits	Health literacy	−.049	.569	[−0.219, 0.124]
Regular visits	Locus of control	−.133	.172	[−0.320, 0.064]
Regular visits	Life not worth living	−.050	.564	[−0.220, 0.123]
Regular visits	Lifetime suicidal thoughts	−.172	.043	[−0.334, 0.000]
Regular visits	Lifetime suicide attempt	−.114	.183	[−0.281, 0.059]
Days between visits	Barthel Index	.094	.305	[−0.091, 0.272]
Days between visits	Existential loneliness (ESTE−R)	−.109	.231	[−0.286, 0.075]
Days between visits	Social loneliness (ESTE−II)	.126	.169	[−0.059, 0.303]
Days between visits	Activities	−.063	.492	[−0.244, 0.122]
Days between visits	Health literacy	.023	.801	[−0.161, 0.205]
Days between visits	Locus of control	.112	.276	[−0.096, 0.310]
Days between visits	Life not worth living	.025	.788	[−0.159, 0.207]
Days between visits	Lifetime suicidal thoughts	.017	.853	[−0.167, 0.199]
Days between visits	Lifetime suicide attempt	−.024	.796	[−0.206, 0.160]
Barthel Index	Existential loneliness (ESTE−R)	−.221	.009	[−0.378, −0.051]
Barthel Index	Social loneliness (ESTE−II)	−.122	.154	[−0.288, 0.051]
Barthel Index	Activities	−.058	.498	[−0.228, 0.115]
Barthel Index	Health literacy	−.153	.072	[−0.316, 0.019]
Barthel Index	Locus of control	.007	.943	[−0.188, 0.201]
Barthel Index	Life not worth living	−.140	.100	[−0.304, 0.032]
Barthel Index	Lifetime suicidal thoughts	−.037	.666	[−0.207, 0.135]
Barthel Index	Lifetime suicide attempt	.029	.731	[−0.143, 0.200]
Existential loneliness (ESTE−R)	Social loneliness (ESTE−II)	.481	<.001	[0.337, 0.603]
Existential loneliness (ESTE−R)	Activities	−.221	.009	[−0.379, −0.051]
Existential loneliness (ESTE−R)	Health literacy	.196	.021	[0.026, 0.356]
Existential loneliness (ESTE−R)	Locus of control	.147	.130	[−0.049, 0.332]
Existential loneliness (ESTE−R)	Life not worth living	.239	.005	[0.071, 0.394]
Existential loneliness (ESTE−R)	Lifetime suicidal thoughts	.192	.024	[0.021, 0.351]
Existential loneliness (ESTE−R)	Lifetime suicide attempt	.070	.412	[−0.102, 0.239]
Social loneliness (ESTE−II)	Activities	−.308	<.001	[−0.456, −0.143]
Social loneliness (ESTE−II)	Health literacy	.252	.003	[0.084, 0.407]
Social loneliness (ESTE−II)	Locus of control	.212	.029	[0.017, 0.391]
Social loneliness (ESTE−II)	Life not worth living	.236	.005	[0.067, 0.392]
Social loneliness (ESTE−II)	Lifetime suicidal thoughts	.219	.010	[0.049, 0.377]
Social loneliness (ESTE−II)	Lifetime suicide attempt	.076	.376	[−0.097, 0.245]
Activities	Health literacy	−.160	.061	[−0.323, 0.012]

Variable 1	Variable 2	ρ	p	95% CI
Activities	Locus of control	−.196	.043	[−0.377, −0.001]
Activities	Life not worth living	.057	.503	[−0.116, 0.227]
Activities	Lifetime suicidal thoughts	.066	.442	[−0.107, 0.235]
Activities	Lifetime suicide attempt	.066	.445	[−0.108, 0.235]
Health literacy	Locus of control	.084	.387	[−0.112, 0.274]
Health literacy	Life not worth living	.084	.323	[−0.088, 0.252]
Health literacy	Lifetime suicidal thoughts	−.082	.337	[−0.250, 0.091]
Health literacy	Lifetime suicide attempt	.092	.280	[−0.080, 0.260]
Locus of control	Life not worth living	.115	.235	[−0.081, 0.303]
Locus of control	Lifetime suicidal thoughts	.207	.032	[0.013, 0.386]
Locus of control	Lifetime suicide attempt	.073	.453	[−0.123, 0.264]
Life not worth living	Lifetime suicidal thoughts	.314	<.001	[0.151, 0.460]
Life not worth living	Lifetime suicide attempt	.194	.022	[0.023, 0.353]
Lifetime suicidal thoughts	Lifetime suicide attempt	.478	<.001	[0.334, 0.600]

Note. ρ = Spearman's rank correlation coefficient; CI = confidence interval; ESTE-II = Social Loneliness Scale; ESTE-R = Revised ESTE Loneliness Scale. Confidence intervals are based on Fisher's z. Binary yes/no variables were coded as 1 = yes and 0 = no. Higher scores indicate greater social loneliness for ESTE-II, greater existential loneliness for ESTE-R, lower health literacy for the health literacy score, and a more external locus of control for the locus of control item.
